# Antimicrobial Susceptibility of Lactic Acid Bacteria Strains of Potential Use as Feed Additives - The Basic Safety and Usefulness Criterion

**DOI:** 10.3389/fvets.2021.687071

**Published:** 2021-07-01

**Authors:** Ilona Stefańska, Ewelina Kwiecień, Katarzyna Jóźwiak-Piasecka, Monika Garbowska, Marian Binek, Magdalena Rzewuska

**Affiliations:** ^1^Department of Preclinical Sciences, Institute of Veterinary Medicine, Warsaw University of Life Sciences, Warsaw, Poland; ^2^Department of Fermentation Technology, Prof. Waclaw Dabrowski Institute of Agriculture and Food Biotechnology – State Research Institute, Warsaw, Poland; ^3^Division of Milk Biotechnology, Department of Biotechnology, Microbiology and Food Evaluation, Institute of Food Sciences, Warsaw University of Life Sciences, Warsaw, Poland

**Keywords:** acquired resistance genes, antimicrobial susceptibility testing, food additives, minimum inhibitory concentration, lactic acid bacteria, probiotics, reservoir of resistance determinants

## Abstract

The spread of resistance to antibiotics is a major health concern worldwide due to the increasing rate of isolation of multidrug resistant pathogens hampering the treatment of infections. The food chain has been recognized as one of the key routes of antibiotic resistant bacteria transmission between animals and humans. Considering that lactic acid bacteria (LAB) could act as a reservoir of transferable antibiotic resistance genes, LAB strains intended to be used as feed additives should be monitored for their safety. Sixty-five LAB strains which might be potentially used as probiotic feed additives or silage inoculants, were assessed for susceptibility to eight clinically relevant antimicrobials by a minimum inhibitory concentration determination. Among antimicrobial resistant strains, a prevalence of selected genes associated with the acquired resistance was investigated. Nineteen LAB strains displayed phenotypic resistance to one antibiotic, and 15 strains were resistant to more than one of the tested antibiotics. The resistance to aminoglycosides and tetracyclines were the most prevalent and were found in 37 and 26% of the studied strains, respectively. Phenotypic resistance to other antimicrobials was found in single strains. Determinants related to resistance phenotypes were detected in 15 strains as follows, the *aph(3*″*)-IIIa* gene in 9 strains, the *lnu*(A) gene in three strains, the *str*(A)-*str*(B), *erm*(B), *msr*(C), and *tet*(M) genes in two strains and the *tet*(K) gene in one strain. The nucleotide sequences of the detected genes revealed homology to the sequences of the transmissible resistance genes found in lactic acid bacteria as well as pathogenic bacteria. Our study highlights that LAB may be a reservoir of antimicrobial resistance determinants, thus, the first and key step in considering the usefulness of LAB strains as feed additives should be an assessment of their antibiotic resistance. This safety criterion should always precede more complex studies, such as an assessment of adaptability of a strain or its beneficial effect on a host. These results would help in the selection of the best LAB strains for use as feed additives. Importantly, presented data can be useful for revising the current microbiological cut-off values within the genus *Lactobacillus* and *Pediococcus*.

## Introduction

Lactic acid bacteria (LAB) strains are important industrial microorganisms, and they have a long history of safe use as feed additives. They are commonly used as probiotics, animal growth biopromoter, as well as bacterial inoculants for forage ensiling to improve not only the quality but also safety of feed (1, 2). Many LAB species are part of the resident microbiota of the gastrointestinal and genitourinary tracts of humans and animals, where they are thought to exert many health-associated beneficial effects (2). Moreover, they have ability to inhibit other microorganisms, including pathogens that cause foodborne diseases or food spoilage (3).

Among the different genera belonging to the LAB group, mainly *Lactobacillus* spp. and *Pediococcus* spp. have been register as gut biota stabilizers and silage additives (4). The interest in the application of pediococci in animal husbandry is gradually increasing due to the improvement of the characteristics and growth abilities of animals that can be achieved with their use (5). They were shown to be effective as probiotics for broiler chickens, laying hens, piglets, fish, crustaceans, and as silage additives (4). Moreover, many strains produce bacteriocins or bacteriocin-like substances that have well-recognized pathogen inhibitory activities (5). Although *Enterococcus* spp. strains as human probiotics remain controversial, in a point of view of the opportunistic and nosocomial infections caused by these bacteria, they are used as silage additives and probiotics for stabilizing the microbial communities of the gastrointestinal tract of animals (4, 6).

Increasing awareness of probiotics and their therapeutic and prophylactic properties constantly encourages the search for new LAB strains, with beneficial health properties and safe for animal consumption. A wide variety of LAB is used in animal nutrition, either directly or as a source of feed additives. Most LAB species are granted the GRAS status (Generally Regarded As Safe) provided by the US Food and Drug Administration (FDA) and within Europe “QPS status (Qualified Presumption of Safety)” notified by European Food Safety Authority (EFSA), The Panel on Biological Hazards (BIOHAZ), which means that they are considered safe for human and animal consumption and for the environment (7).

Despite that LAB species are widely used and recognized as safe food and feed additives, the rare cases of serious infections in humans caused by LAB have been described in the literature, including bacteremia (8–11), endocarditis (12, 13), pleuropneumonia (8, 14), meningitis (15), and urinary tracts infections (16). The infections occur mainly in patients with serious underlying illnesses, the immunocompromised ones, premature newborns, or elderly individuals. In case of *Lactobacillus* spp. most of the reported clinical cases are related to *Lactobacillus rhamnosus*. Infections associated with *Lactococcus* spp. are mainly concerned to *Lactobacillus lactis* subsp. *lactis* and *Lactobacillus garvieae*, while infections caused by *Pediococcus* spp. and *Leuconostoc* spp. have rarely been described (17, 18). Little is known about the role of LAB in animal infections, although the genus *Lactococcus* may be associated with bovine mastitis and infections in fish and birds (19), up to date there are no reports of *Lactobacillus* and *Pediococcus* infections in animals.

The second serious concern is acquired resistance to antimicrobials of human and veterinary importance among LAB strains (20). There has been increasing attention to this phenomenon since LAB are considered as a reservoir of resistance genes that can be transferred to pathogenic bacteria, leading to the spread of antibiotic resistance among pathogens and complicating the treatment of infection caused by these bacteria (19). Therefore, caution is needed in selecting and monitoring potentially probiotic strains, and antimicrobial resistance (AMR) is regarded as a crucial safety issue during assessing and approving LAB as feed additives (21). The safety assessment of microbial feed additives is governed under specific EU regulatory frameworks in accordance with Regulation (WE) No 1831/2003 and Commission Regulation (EC) No 429/2008. The Panel on Additives and Products or Substances used in Animal Feed (FEEDAP) provides the scientific opinion on the efficacy of feed additives and their safety to target animals, the consumers of products derived from animals treated with the additives, and to the environment. In line with the FEEDAP recommendation, any bacterial strain carrying an acquired gene conferring AMR or strains with the unknown genetic nature of a demonstrated resistance to antimicrobial agents should not be used as a feed additive due to the greatest risk of horizontal spread (21).

The aim of the present study was an AMR safety assessment of selected LAB strains intended for use as feed additives by phenotypic screening of resistance to clinically relevant antimicrobials. The identification of resistance determinants in the resistant LAB strains was also performed in order to exclude the presence of potentially transferable AMR genes.

## Materials and Methods

### Bacterial Strains

The study provides a safety assessment of 65 LAB strains potentially useful as probiotics and other feed additives. Fifty-seven *Lactobacillus* strains [*Lactobacillus plantarum* (*n* = 26), *Lactobacillus fermentum* (*n* = 7), *Lactobacillus casei* (*n* = 3), *L. rhamnosus* (*n* = 3), *Lactobacillus reuteri* (*n* = 3), *Lactobacillus brevis* (*n* = 3)*, Lactobacillus buchneri* (*n* = 2), *Lactobacillus salivarius* (*n* = 2), *Lactobacillus agilis* (*n* = 2), *Lactobacillus acidophilus* (*n* = 1), *Lactobacillus johnsonii* (*n* = 1), *Lactobacillus diolivorans* (*n* = 1), *Lactobacillus delbrueckii* (*n* = 1), *Lactobacillus paracasei* (*n* = 1), *Lactobacillus farraginis* (*n* = 1)], six *Pediococcus* strains [*Pediococcus pentosaceus* (*n* = 5), *Pediococcus acidilactici* (*n* = 1)], and two *Enterococcus* strains [one *Enterococcus durans* strain and one *Enterococcus faecium* strain] were selected for this study (Supplementary Table 1). A total of 47 strains are available at the culture collections: 42 strains at the Collection of Industrial Microbial Cultures (KKP), located at the prof. Waclaw Dabrowski Institute of Agricultural and Food Biotechnology (IAFB) in Warsaw (Poland), four strains at the Polish Collection of Microorganisms (PCM), located at the Institute of Immunology and Experimantal Therapy in Wroclaw (Poland) and one strain from American Type Culture Collection (ATCC). The rest 18 strains were isolated from fermented or fresh vegetables and fruits (*n* = 14) or probiotic drinks (*n* = 4). The isolates were identified by nucleotide sequence analysis of the gene encoding 16S rRNA. LAB strains belonging to the *L. plantarum* phylogenetic group (*L. plantarum, Lactobacillus pentosus*, and *Lactobacillus paraplantarum*) were differentiated by multiplex PCR using species-specific primers amplified the fragment of the *recA* gene encoding the recombinase A (22). The strains isolated from the same sources were typed by RAPD-PCR (Random Amplified Polymorphic DNA) with primers RP and PRIMO2 (23) in order to confirm their intraspecies diversity (data not shown). All strains were stored in a liquid nitrogen atmosphere in MRS (deMan- Rogosa-Sharpe) broth (Oxoid) supplemented with glycerol (15% v/v). Before the antibiotic susceptibility assay, LAB strains were cultivated in MRS agar (Oxoid) at 37°C for 24–48 h in 5% CO_2_. After incubation, the colonies were suspended in 0.85% NaCl solution to prepare the inoculum for the broth microdilution test.

### Phenotypic Antimicrobial Resistance

The following antimicrobials, used in therapy of common infections, were tested: gentamicin (0.125–64 mg/L), kanamycin (0.5–256 mg/L), streptomycin (0.5–256 mg/L), tetracycline (0.125–64 mg/L), chloramphenicol (0.06–32 mg/L), ampicillin (0.015–8 mg/L), erythromycin (0.015–8 mg/L), and clindamycin (0.015–8 mg/L). Gentamicin, kanamycin, erythromycin, clindamycin originated from the European Pharmacopoeia (EP) Reference Standards, while streptomycin, tetracycline, chloramphenicol, and ampicillin from Sigma-Aldrich. LSM broth (IsoSensitest broth (90%) and MRS broth (10%), adjusted to pH 6.7) and the microdilution method according to Klare et al. (24) were used. The lowest concentration of each antibiotics that inhibits the visible growth of bacteria (MIC, Minimum Inhibitory Concentration) was determined after 48 h of incubation at 37°C and in the presence of 5% CO_2_. Susceptibility of strains was established in accordance with the microbiological cut-off values defined by the EFSA Panel on Additives and Products or Substances used in Animal Feed (21). The accuracy of antimicrobial susceptibility testing was monitored by parallel use of the reference strains, *Enterococcus faecalis* ATCC 29212 and *Escherichia coli* ATCC 25922 as a quality control. The study was performed in triplicate. The differences of MICs for independent sample never exceed 1 order of dilution.

### Genetic Determinants of Antimicrobial Resistance

All LAB strains phenotypically resistant to the tested antimicrobial agents were examined by PCR for the presence of selected AMR genes. The following genes were detected: *bla* gene (ampicillin-resistant strains); the *erm*(A), *erm*(B), *erm*(C), *msr* genes, and the *lnu*(A) gene (erythromycin and/or clindamycin-resistant strains); genes encoding ribosomal protection proteins (universal primer set and subsequently, specific primers for *tet*(W) and *tet*(M) genes for positive strains) and the *tet*(K) and *tet*(L) genes encoding a tetracycline efflux pump (tetracycline-resistant strains); the *cat* gene (chloramphenicol-resistant strains); the *aph*(3″)*-IIIa* gene (kanamycin-resistant strains); the *ant*(6), *str*(A)/*str*(B) and *aad*(A) genes (streptomycin-resistant strains); the *aac*(6′)-*aph*(2″) gene (aminoglycosides-resistant strains). In case of the detection of resistance genes, the cut-off values given in the previous EFSA guidance (25) were additionally used for a results analysis.

The characteristics of the primers used in the study and appropriate references (26–36) are shown in Supplementary Table 2. The primer set for *msr*(C) detection was designed using the PCR Primer Design Tool (https://eurofinsgenomics.eu/en/ecom/tools/pcr-primer-design) and checked using an Oligo Analysis Tool (https://eurofinsgenomics.eu/en/ecom/tools/oligo-analysis). PCR reactions were performed in a total volume of 25 μL containing 1 μL of each primer (10 pmol/μL), 12.5 μL of DreamTaq PCR Master Mix (2×) (ThermoFisher Scientific) or JumpStart REDTaq ReadyMix Reaction Mix (2×) (Sigma-Aldrich) and 50 ng of DNA template. A template bacterial genomic DNA was purified using GenElute™ Bacterial Genomic DNA Kits (Sigma-Aldrich) following the manufacturer's instruction for Gram-positive bacteria cells (pre-incubation with lysozyme). The amount and quality of DNA was determined using the Thermo Scientific NanoDrop^TM^ 1000 Spectrophotometer.

PCR products were separated by electrophoresis on a 1% agarose gel (Sigma-Aldrich), stained with ethidium bromide, in TBE buffer (100 V). The O'RangeRuler^TM^ 200bp DNA Ladder, GeneRuler^TM^ 100 bp DNA Ladder or GeneRuler^TM^ 100 bp Plus DNA Ladder (ThermoFisher Scientific) were used as size standard markers. Additionally, PCR products were purified and sequenced (Genomed S.A.). The obtained DNA sequences were analyzed using BLASTn (Basic Local Alignment Search Tool, http://blast.ncbi.nlm.nih.gov/Blast.cgi) and compared with sequences available in GenBank (National Center for Biotechnology Information) and CARD database (The Comprehensive Antibiotic Resistance Database, https://card.mcmaster.ca) (Supplementary Table 3).

### Nucleotide Sequence of AMR Genes

The nucleotide sequences of the *msr*(C), *erm*(B), *lnu*(A), *aph*(3″)*-IIIa, str*(B), *tet*(M), and *tet*(K) genes described in this study are shown in Supplementary Table 4.

## Results

### Phenotypic Antimicrobial Resistance

Each strain was able to grow on LSM medium without antibiotic (growth positive control). The MICs of antibiotics for studied strains are presented in Table 1 and Supplementary Table 5. The MIC ranges for particularly antibiotics were varied and were within the used concentration ranges of tested antibiotics: for gentamicin <0.125–32 mg/L, for kanamycin 4–≥256 mg/L, for streptomycin <0.5–≥256 mg/L, for tetracycline 0.25–32 mg/L, for chloramphenicol 1–8 mg/L, for ampicillin <0.015–≥8 mg/L, for erythromycin <0.015–≥8 mg/L, and for clindamycin <0.015–≥8 mg/L (Table 2). Only 31 strains (17 *L. plantarum*, four *L. fermentum* and *L. casei*, three *L. reuteri* and one *L. buchneri, L. agilis* and *L. rhamnosus*) out of 65 strains were susceptible to all antibiotics as the microbiological cut-off values were below the proposed by the FEEDAP Panel breakpoints (21). Nineteen LAB strains were resistant to one of the investigated antibiotics (i.e., 11 strains to kanamycin, seven to tetracycline and one to ampicillin), whereas 15 strains displayed resistance to more than one of the investigated antibiotics (i.e., 8 strains to two antibiotics, 6 strains to three and one strain to four antibiotics) (Tables 1, 3). The resistance to aminoglycosides was the most prevalent (37%), since 21 strains (32%) were resistant to kanamycin, 10 to streptomycin (15%) and two to gentamicin (3%). The *Lactobacillus agilis* KKP 1834 strain was highly resistant to all aminoglycosides tested, as the MIC values were twice higher than the corresponding breakpoints proposed by the EFSA. The tetracycline resistance was the second common antibiotic resistance found in the studied LAB strains, and was reported in 17 resistant strains (26%). The resistance to erythromycin or chloramphenicol was reported in two strains, while single strains were phenotypically resistant to clindamycin or ampicillin. The MIC, MIC_50_, and MIC_90_ values of tested antibiotics for all studied strains are shown in Tables 1, 2.

**Table 1:** Distribution of MICs of tested antibiotics among phenotypically resistant LAB strains (*n* = 34).

**Number**	**Strain**	**MIC (mg/L)^a^**							
		**GM^**b**^**	**K**	**TE**	**CH**	**A**	**E**	**CL**	**S**
**Microbiological cut-off values (mg/L) proposed by EFSA for obligate heterofermentative** ***Lactobacillus***									
		16	64(32)^**c**^	8	4	2	1	4(1)	64
1	*L. buchneri* KKP 2047p	4	**128**	16^d^	4	1	0,25	0,5	64
2	*L. diolivorans* KKP 2036p	8	**128**	**16**	2	2	0,25	0,125	**128**
3	*L. fermentum* KKP 2020	2	32	**16**	4	0,5	0,25	0,25	32
4	*L. fermentum* KKP 830	8	64	**16**	2	1	0,25	0,25	64
5	*L. fermentum* Sieger	16	**128**	4	4	0,125	0,125	0,03	32
6	*L. brevis* Pap3/4	2	64	**16**	4	0,25	0,125	0,25	64
7	*L. brevis* Pat1	0,5	16	**16**	4	2	0,5	≤0,015	8
8	*L. brevis* Solaris	1	16	**16**	4	1	0,25	0,5	16
9	*L. farraginis* E/J	0,5	8	**16**	4	0,125	0,03	0,03	8
**Microbiological cut-off values (mg/L) proposed by EFSA – facultative heterofermentative** ***Lactobacillus***									
		16	64	8	4	4	1	4(1)	64
10	*L. agilis* KKP 1834	**32**	**≥256**	0,25	4	1	**≥8**	1	**≥256**
11	*L. salivarius* KKP 1828	16	**128**	1	4	0,25	0,125	0,125	**128**
12	*L. salivarius* KKP 1835	8	**128**	**16**	4	2	0,125	0,03	**128**
**Microbiological cut-off values (mg/L) proposed by EFSA for** ***Lactobacillus rhamnosus***									
		16	64	8	4	4	1	4(1)	32
13	*L. rhamnosus* KKP 849	4	**128**	1	**8**	1	0,5	0,5	32
14	*L. rhamnosus* B/J	**32**	**128**	1	4	0,5	0,125	0,5	32
**Microbiological cut-off values (mg/L) proposed by EFSA for** ***Lactobacillus plantarum/pentosus***									
		16	64	32	8	2	1	4(2)	n.r.
15	*L. plantarum* KKP 804	4	64	32	4	**≥8**	0,25	4	n.r.
16	KKP 815	8	**128**	16	8	1	0,25	2	n.r.
17	KKP 835	8	**≥256**	16	8	2	0,25	1	n.r.
18	KKP 870	16	**≥256**	32	8	2	0,25	4	n.r.
19	KKP 872	16	**≥256**	16	8	2	0,25	4	n.r.
20	KKP 2021p	4	**128**	16	8	1	0.25	4	n.r.
21	KKP 1821	4	**128**	16	4	1	0,25	0,5	n.r.
22	KKP 1822	8	**128**	16	8	1	0,25	0,5	n.r.
23	ATTC 8287	8	**128**	16	8	2	0,5	2	n.r.
**Microbiological cut-off values (mg/L) proposed by EFSA for obligate homofermentative** ***Lactobacillus***									
		16	16	4	4	2(1)	1	4(1)	16
24	*L. delbrueckii* PCM 490	4	**32**	2	2	0,06	0,06	0,06	8
**Microbiological cut-off values (mg/L) proposed by EFSA for** ***Lactobacillus acidophilus*** **group**									
		16	64	4	4	1	1	4(1)	16
25	*L. acidophilus* PCM 2499	4	16	**32**	2	0,25	1	0,125	**32**
26	*L. johnsonii* KKP 878	4	64	**16**	**8**	0,125	0,25	0,5	**32**
**Microbiological cut-off values (mg/L) proposed by EFSA for** ***Pediococcus*** **spp**.									
		16	64	8	4	4	1	1	64
27	*P. pentosaceus* KapA	4	**128**	**16**	4	2	0,25	0,03	**128**
28	*P. pentosaceus* Pom7	4	64	**16**	2	1	0,25	0,03	64
29	*P. pentosaceus* AG	16	**128**	**16**	4	2	0,5	0,03	**128**
30	*P. pentosaceus* MA	16	**≥256**	**16**	4	2	0,25	0,03	64
31	*P. pentosaceus* WN1	8	64	**16**	4	1	0,5	0,03	**128**
32	*P. acidilactici* KKP 1839	4	**128**	**16**	4	2	0,25	0,03	**128**
**Microbiological cut-off values (mg/L) proposed by EFSA for** ***Enterococcus*** **spp**.									
		32	1024	4	16	2	4	4	128
33	*E. durans* KKP 1586	16	64	0,5	8	0,25	**≥8**	**≥8**	128
34	*E. faecium* TR2	32	128	**32**	2	0,125	**≥8**	4	128

a *MICs higher than EFSA cut-off values in bold*;

b *GM, gentamicin; K, kanamycin; TE, tetracycline; CH, chloramphenicol; A, ampicillin; E, erythromycin; CL, clindamycin; S, streptomycin*;

c *the previous EFSA proposed cut-off values (2012) are given in brackets*;

d *L. buchneri the cut-off for tetracycline is 128; KKP - strains from the Culture Collection of Industrial Microorganisms; PCM - strains from The Polish Collection of Microorganisms; n.r., not required*.

**Table 2:** Distribution of the MIC, MIC_50_, and MIC_90_ values of eight antibiotics among studied LAB species (*n* = 65).

**Antibiotic**	**MIC values (mg/L)**																
	**0,015**	**0,003**	**0,06**	**0,0125**	**0,25**	**0,5**	**1**	**2**	**4**	**8**	**16**	**32**	**64**	**128**	**256**	**MIC_**50**_**	**MIC_**90**_**
Gentamicin				1		3	8	10	19	16	7	3				4	16
Kanamycin									2	1	8	14	21	16	5	64	128
Streptomycin^a^						1			2	4	6	11	6	9	1	32	128
Tetracycline					2	2	8	3	5	3	37	7				16	32
Erythromycin	1	2	4	20	30	5	2			3						0,25	1
Clindamycin	6	13	5	7	9	14	4	3	5	1						0,25	2
Ampicillin	1	1	3	14	10	5	17	14	1	1						0,5	2
Chloramphenicol							2	13	34	18						4	8

a *27 L. plantarum strains were not tested*.

**Table 3:** Correlation between resistance phenotype and genotype among studied LAB species (*n* = 40).

**Strains**	**Resistance phenotype^**a**^**	**Resistance genotype**
*L. fermentum* KKP 2020	TE	n.d.
*L. fermentum* KKP 830		n.d.
*L. brevis* Pat1		n.d.
*L. brevis* Solaris		n.d.
*L. brevis* Pap3/4		n.d.
*L. farraginis* E/J		n.d.
*P. pentosaceus* Pom7		n.d.
*L. buchneri* KKP 2047p	K	*aph*(3″)*-IIIa*
*L. fermentum* Sieger		n.d.
*L. plantarum* KKP 815		n.d.
*L. plantarum* KKP 835		*aph*(3″)*-IIIa*
*L. plantarum* KKP 870		*aph*(3″)*-IIIa*
*L. plantarum* KKP 872		n.d.
*L. plantarum* KKP 2021p		n.d.
*L. plantarum* KKP 1821		n.d.
*L. plantarum* KKP 1822		*aph*(3″)*-IIIa*
*L. plantarum* ATCC 8287		n.d.
*L. delbrueckii* PCM 490		n.d.
*P. pentosaceus* MA	K – TE	n.d.
*L. salivarius* KKP 1828	K – S	n.d.
*L. rhamnosus* KKP 849	K – CH	n.d.
*L. plantarum* KKP 804	A	n.d.
*L. rhamnosus* B/J	GM – K	n.d.
*L. acidophilus* PCM 2499	TE – S	*tet*(K), *str*(A)/*str*(B)
*P. pentosaceus* WN1		n.d.
*E. durans* KKP 1586	E – CL	*erm*(B)
*E. faecium* TR2	TE – E	*tet*(M), *msr*(C), *lnu*(A)
*L. diolivorans* KKP 2036p	TE – K – S	*aph*(3″)*-IIIa*
*L. salivarius* KKP 1835		*tet*(M), *str*(A)/*str*(B)
*P. pentosaceus* KapA		n.d.
*P. pentosaceus* AG		n.d.
*P. acidilactici* KKP 1839		n.d.
*L. johnsonii* KKP 878	TE – CH – S	n.d.
*L. agilis* KKP 1834	GM – K – S – E	*aph*(3″)*-IIIa, msr*(C)
***L. fermentum*** **KKP 811, KKP 830, KKP 843**	K	*aph*(3″)*-IIIa*
***L. plantarum*** **KKP 870, KKP 872**	CL	*lnu*(A)
***L. plantarum*** **KKP 2021p**	CL	*erm*(B)

a *GM, gentamicin; K, kanamycin; TE, tetracycline; CH, chloramphenicol; A, ampicillin; E, erythromycin; CL, clindamycin; S, streptomycin; n.d., tested resistance genes not detected. The strains carrying a resistance gene but phenotypically resistant only in line to cut-off values adopted in previous EFSA guideline (2012) are in bold*.

### Distribution of AMR Genes

To identify determinants responsible for the displayed resistance phenotypes, the strains were screened by PCR for the presence of selected AMR genes. Acquired AMR genes were only found in 15 strains (Table 3). When investigating 17 tetracycline-resistant strains, the *tet*(M) gene encoding ribosomal protection proteins were found in two strains (*L. salivarius* KKP 1835 with tetracycline MIC value of 16 mg/L and *E. faecium* TR2 with MIC value of 32 mg/L). *L. acidophilus* 2499 strain displaying the MIC value of tetracycline three times higher than the breakpoint (32 vs. 4 mg/L), was positive for the *tet*(K) gene. The *erm*(B) gene was detected in *L. plantarum* KKP 2021p (the MIC value of clindamycin was 4 mg/L, but the strain was susceptible to erythromycin, MIC = 0.25 mg/L) and in *E. durans* KKP 1586 (erythromycin and clindamycin MIC values were 8 mg/L and higher than 8 mg/L, respectively). In addition, two *L. plantarum* strains resistant to clindamycin (870 and 872, with MIC value 4 mg/L) and *E. faecium* TR2, susceptible to clindamycin, carried the *lnu*(A) gene. Two strains were positive for the *msr*(C) gene, *L.agilis* KKP 1834 and *E. faecium* TR2 strains (erythromycin MIC value was 8 mg/L). The *aph(3*″*)-IIIa* gene was detected in 9 strains belonging to the species: *L. plantarum* (*n* = 3), *L. fermentum* (*n* = 3), *L. buchneri* (*n* = 1), *L. diolivorans* (*n* = 1), and *L. agilis* (*n* = 1). Two strains, *L. acidophilus* 2499 and *L. salivarius* 1835, with streptomycin MIC values 32 and 128 mg/L, respectively, were positive for *str*(A)/*str*(B) genes.

The selected PCR amplicons were sequenced, and the obtained sequences of the tested AMR genes (Supplementary Table 4) indicates the homology to the DNA sequences detected in other LAB, as well as in pathogens (Supplementary Table 3). The PCR product for *msr*(A)/*msr*(B) genes, encoding for a macrolide efflux protein and conferring resistance to macrolides and streptogramins B, were identified as the *msr*(C) gene by sequencing (Supplementary Table 3). No specific primers targeting the *msr*(C) gene were found in the available literature, thus we designed a primer set to detect this gene without the need for sequencing of the PCR product. For both strains, *L*. *agilis* KKP 1834 and *E. faecium* TR2, the specific product of 354-bp with newly designed primer set was obtained. In the case of ampicillin resistant strains, a product of ~297 bp obtained with primers specific for the *bla* gene was found in one strain (*L. plantarum* 804). However, the presence of this gene is questionable as the chromatograms obtained by sequencing were unreadable despite the repetition.

## Discussion

It is generally accepted that starter cultures or feed additives contain strains isolated from target raw materials, in accordance with their intended use. The source of probiotic strains used in animals are often the gastrointestinal tract or feces of the same or different animal species (37). Natural microbiota isolated from the host usually more easily and quickly adapts and could be more effective as a probiotic compared to strains from other sources. Nevertheless, numerous studies indicate high prevalence of drug resistance in strains isolated from various animals, including pigs, ruminants, companion animals, poultry, or even wild animals (38–41) as well as from food of animal origin (30, 42). The intensive and irresponsible (especially non-therapeutic) use of antimicrobial agents in animal husbandry and veterinary practice contributes to developing of resistance of gut microbiota and potentially beneficial LAB to antibiotics, including tetracycline, enrofloxacin, ampicillin and MLS antibiotics (macrolides, lincosamides and streptogramins) (20, 40, 41, 43, 44). Such strains considered as a reservoir of AMR genes for other commensal bacteria, as well as pathogenic and opportunistically pathogenic species through horizontal gene transfer (20, 45). This poses a threat not only to animals, but resistant strains can also be widely distributed through the food chain. Hence, the use of LAB strains isolated from non-intestinal sources has become increasingly attractive and justified. The alternative sources from which beneficial LAB can be isolated are fruits, vegetables and juices, cereals, silages, sourdough, fermented foods and beverages, as well as raw materials and ingredients used to make non-fermented and fermented foods (37, 46). The strains selected from various “unconventional” sources meet the criteria for probiotic strains, such as resistance to low pH and high bile concentrations, adherence capacity to epithelial intestinal cells, and strong antimicrobial activity against pathogenic microorganisms, including bacteriocin-like activity (37). The strains deposited in different Microbial Culture Collections can also be screened to find beneficial LAB strains, although this does not appear to be a common practice. The advantage of strains from the Collections with the status of International Deposit, however, may be their widespread availability. In the present study we used LAB strains from both sources, isolated from animal origin and strains from alternative sources. Most of the strains are deposited in the Microbial Culture Collections.

Recently, the taxonomy of genus *Lactobacillus* changed significantly. The genus *Lactobacillus* was one of the most taxonomically complex and extremely heterogeneous and composed 261 genera (as of March 2020) (47). In 2020, based on polyphasic approach (phylogenomic analysis), Zheng et al. (47) reclassified the genus *Lactobacillus* into 25 genera, including 23 new one. The emended genus of *Lactobacillus* currently consists of 38 species well adapted to vertebrates' or invertebrates' hosts. The general term lactobacilli are further used to designate bacteria classified to the family *Lactobacillaceae* until 2020. In our work, we use the names of the former *Lactobacillus* classification to avoid any confusion and for maintenance of compliment with the nomenclature used in EFSA guidance for microbiological cut-off values. It should be highlighted that the complexity of this phylogenetic group of microorganisms make it difficult to generalize about this genus and contributes to many difficulties in antimicrobial susceptibility testing of these bacteria, regarding the appropriate medium or establish the cut-off values.

LAB species differ significantly in their growth requirements. The M45 (3rd ed.) CLSI (Clinical Laboratory Standards Institute) procedure proposes the use of cation-adjusted Mueller-Hinton broth (CAMHB) supplemented with 2.5 or 5% lysed horse blood (LHB) as a conventional susceptibility test medium, however, some lactobacilli exhibited weak growth in this medium (24, 48). In this study, we used the LSM broth proposed by Klare et al. (24) and in line with ISO/IDF standard procedure, which is more accurate and reproducible for lactobacilli and pediococci (24, 48). To distinguish strains with phenotypic resistance from susceptible one, the MIC-off value proposed by the EFSA FEEDAP were used (21). The standard procedures of the European Committee on Antimicrobial Susceptibility Testing (EUCAST) and CLSI provide the same breakpoints for all lactobacilli species, while the EFSA's guidelines refer to different groups within LABs, which is relevant considering the great differences in AMR among lactobacilli species. Some species of *Lactobacillus* are intrinsically resistant to certain antibiotics (e.g., *L. plantarum/L. pentosus* to streptomycin), while other lactobacilli have variable activity against these antimicrobials (49). Moreover, the breakpoint values are best established for clinically important microorganisms. In the case of lactobacilli, which are infrequently associated with a clinical infection, the collected data are limited, and the guidelines of CLSI and EUCAST provide breakpoints for only four of antibiotics testing (ampicillin, clindamycin, chloramphenicol, and erythromycin).

Antimicrobial susceptibility is a key criterion that must be met when microorganisms are intentionally introduced into the food chain. Numerous data indicate that LAB exhibit highly variable sensitivity to antimicrobial agents. In our study, a total of 65 strains intended for use as a feed or silage additives were tested for their susceptibility to eight selected antimicrobials. Thirty-four tested strains were resistant to at least one antimicrobial agent according to a current EFSA guidance (21).

The high susceptibility of LAB strains to ampicillin (98.5%) was observed in our study, which is in line with a number of previous data (29, 50, 51). However, it should be noted that higher resistance for this antibiotic was also noted in lactobacilli, mainly in isolates from poultry and fermented dairy products (40, 41, 52). The resistance to β-lactam antibiotics is related to the presence of the *bla* gene whilst we not confirmed by sequencing the presence of this gene in ampicillin-resistant *L. plantarum* strain. The absence of genes associated with β-lactam resistance among strains with relatively high MIC values was observed by others (40, 41).

High susceptibility among tested LAB strains has been also noted in case of chloramphenicol (96.9%). This is consistent with many published data (40, 50, 51, 53), although resistance to chloramphenicol in lactobacilli strains isolated from various fermented products has also been reported (52, 54). The genotypic resistance to this antibiotic class is usually associated with the presence of *cat* gene (55) and the occurrence of this gene was noted among some of LAB strains, including *L. salivarius, L. johnsonii, L. crispatus, L. reuteri, L. plantarum, L. ingluviei*, and *P. acidilactici* (40, 41, 54). Interestingly, the *cat* gene was not detected in chloramphenicol-resistant *L. rhamnosus* and *L. johnsonii* strains in this study (MIC = 8 mg/L while the cut-off values is 4 mg/L). According to the literature data, the resistance to chloramphenicol may not be related only to the presence of specific genes encoding antibiotic-modifying enzymes, but may also result from diminished expression of many genes, including efflux pumps and oxidative stress-related genes as well as genes encoding outer membrane proteins (56). This phenomenon may be a cause of phenotype and genotype inconsistency observed also in the tested strains.

The occurrence of tetracycline resistance was found in 26.2% of LAB strains in this study. In other studies conducted in Poland, the percentage of tetracycline-resistant lactobacilli was significantly higher (40, 41, 53), however, it is not surprising considering that these strains were isolated from poultry. The *tet* genes are often found in isolates of animal origin (38, 39), while in lactobacilli strains isolated from fermented food the resistance to tetracyclines is less frequent, like our findings (29, 52). The prevalence of the *tet* genes which confers resistance to teracyclines was not significant among tested LAB strains. The *tet*(M) gene encoded the ribosomal protection protein was found in *L. salivarius* and *E. faecium* strains whilst *tet*(K) encoded the energy-dependent efflux protein was presented in *L. acidophilus*. Similarly, the *tet*(M) gene was noted in *E. faecium* and *L. salivarius* isolates from fermented food in India (57). Nawaz et al. (29) detected this gene in *L. plantarum, L. salivarius, L. animalis*, and *L. brevis* strains isolated from fermented food. This gene was also widespread in *L. salivarius, L. agilis*, and *L. crispatus* strains isolated from chickens, turkeys, and pigeons in Poland (40, 41, 53). Generally, the *tet*(M) gene is one of the most widespread tetracycline resistance determinants in lactobacilli (55). The *tet*(K) gene has so far been detected in strains of *L. fermentum, L. buchneri*, and *P. pentosaceus* from fermented food (51, 57) or *L. plantarum, L. salivarius*, and *L. reuteri* isolates from meat pork and poultry in Italy (42). Interestingly, to the best of our knowledge, it seems that *tet*(K) has not been previously described in *L. acidophilus*. Among the LAB strains tested, we observed the highest prevalence of phenotypic tetracycline resistance in obligate heterofermentative lactobacilli (64% strains) and pediococci (100% strains) (MIC = 16 mg/L), but *tet* resistance genes were not detected in any of the strains. Similar results were reported by other authors (40, 41, 58). This contradiction between the phenotypic resistance and the absence of the *tet* genes indicates that tetracycline resistance in these bacteria is likely to be intrinsic and the current microbiological cut-off values for tetracycline should be reevaluated. We propose the MIC = 16 mg/L as cut-off value for categorization of susceptible and resistant strains within obligate heterofermentative *Lactobacillus* spp. and *Pediococcus* spp. The pediococci resistance to tetracyclines was considered as intrinsic also by other authors, who failed to detect the *tet* genes in strains with MIC values ≥16 mg/L (32, 58–60). The high resistance to tetracycline that may be naturally conditioned was also discussed in lactobacilli species (50, 61). The intrinsic resistance to tetracyclines is related to the complex regulatory network that modulate the uptake, as well as intracellular accumulation of these antibiotics. The mutations affect to expression and function of activator or repressor of pumps and porins (62). The regulation of intrinsic tetracycline resistance is better characterized in Gram-negative bacteria. The available data about this resistance in Gram-positive species are still poorly understood.

The low rates of resistance to erythromycin (4.6%) and clindamycin (1.5%) were observed in tested LAB strains, although other reports showed the high prevalence of resistance to these antimicrobials among lactobacilli strains (40, 41, 43, 44, 52). The *erm*(B) gene encoding the ribosomal RNA methylase was detected in *L. plantarum* and *E. durans*. The presence of the *erm* genes is related to exhibit of MLS_B_ resistance phenotype (macrolides-lincosamides-streptogramins B), however, only *E. durans* 1586 was resistant to erythromycin and clindamycin, whereby *L. plantarum* 2021p was susceptible to both antimicrobials. It is also worth highlighted that the recommendation for clindamycin has been revised and the current cut-off value for all lactobacilli is MIC = 4 mg/L (21). According to the previous guidance (25), this strain would be considered phenotypically resistant to clindamycin, however still susceptible to erythromycin. The presence of the *erm* genes in strains with phenotypic susceptibility to MLS or only erythromycin was previously reported by others (40, 43, 44) and may be related to defective expression of this gene (43, 44). The relatively high occurrence of *erm*(B) was noted for different *Lactobacillus* and *Enterococcus* strains isolated from fermented food (29, 57). The *erm*(B) gene was detected in different lactobacilli (*L. plantarum, L. jonsonii, L. salivarius, L. reuteri, L. crispatus, L. amylovorus, L. gallinarum*) isolated from broilers (43, 44), from swine and poultry meat products (42) or from wine (59). Moreover, in our study two erythromycin-resistant strains, *L. agilis* 1834 and *E. faecium* TR2, carried the *msr*(C) gene. To the best of our knowledge, this is the first study which reports the presence of this gene in *L. agilis*. The *msr*(C) gene was initially considered as characteristic for *E. faecium* (63), then it was found in other *Enterococcus* species, including *E. durans, E. lactis*, and *Enterococcus casseliflavus*, and also *P. pentosaceus* and *L. fermentum* strains (57). The ever frequently occurrence of *msr*(C) in different LAB species may be associated with increasingly widespread transfer of this gene between these bacteria. Moreover, two *L. plantarum* (870, 872) and one *E. faecium* TR2 strains, phenotypically susceptible to clindamycin with MIC = 4 mg/L, carried the *lnu*(A) gene which encoding lincosamide *O*-nucleotidyltransferase. This gene was found in *L. salivarius, L. johnsonii, L. crispatus, L. reuteri, L. agilis*, and *L. ingluviei* (40, 53). Similarly, to our results, also Dec et al. (53) noted the *lnu*(A) gene in lactobacilli strains susceptible to clindamycin. However, the reason of this relationship remains unknown. In the other hand, the presence of *lnu*(A) gene in strains with the clindamycin MIC of 4 mg/L may suggest that the previous cut-off values (25) were more suitable to distinguish between a susceptible and a resistant strain. Interestingly, it seems that according to available data *lnu*(A) has not been described so far in *L. plantarum* and *E. faecium* species.

In the current study, we observed a high resistance of LAB strains to kanamycin (32.3%) and streptomycin (15.4%), while gentamicin resistance was much less prevalent (3.1%). Similarly, more frequent occurrence of resistance to streptomycin than to gentamicin was recorded for lactobacilli from chickens and turkeys in Poland (40, 41). However, the higher resistance to gentamicin was also reported previously (52). The widespread occurrence of kanamycin-resistant lactobacilli strains of various species has been noted by others (29, 51, 53). It is generally known that some lactobacilli species display resistance to aminoglycosides. Of the genes that determine resistance to aminoglycosides, the most prevalent was *aph*(3″)*-IIIa*, encoding the kinase APH(3″)-IIIa, which confer resistance to kanamycin. This gene was found in 6 kanamycin-resistant strains with the MIC value in the range from 128 to ≥256 mg/L, including *L. plantarum* (835, 870, 1822), *L. buchneri* 2047p, *L. diolivorans* 2036p, and *L. agilis* 1834. The *aph*(3″)*-IIIa* gene has been previously detected in *L. delbrueckii* subsp. *bulgaricus* and *Streptococcus thermophilus* strains from yogurts (64) and *L. plantarum* isolated from wine (59). Surprisingly, the presence of *aph*(3″)*-IIIa* was also noted in this study in three *L. fermentum* strains (811, 830, 843) with MIC = 64 mg/L, classified as susceptible to kanamycin. Similarly, to our results, the presence of aminoglycoside resistance genes in phenotypically susceptible lactobacilli have been observed previously (40). Moreover, the *str*(A)*/str*(B) genes, encoding the streptomycin kinases APH(3″)-Ib and APH(6′)-Id, respectively, were noted in *L. acidophilus* 2499 and *L. salivarius* 1835. Interestingly, both these strains had MIC values on-fold higher than the cut-off value for streptomycin (32 mg/L for *L. acidophilus* 2499 and 128 mg/L for *L. salivarius* 1835). It should be highlighted, that the *str*(A) and *str*(B) genes are most frequently linked (65). In this study, we used the primer set which can detect both these genes, whereby a primer forward is complementary to the final part of the *str*(A) gene. Therefore, the partial sequence of *str*(B) is the main PCR product. The possible occurrence of *str*(A) should be confirmed by additional sequencing of longer fragments of this gene or using a specific primer set. It should be mentioned that the vast majority of phenotypically aminoglycoside-resistant strains did not contain any of the known genes that determine this resistance. This phenomenon has been described in other reports (53) and it was suggested that resistance to aminoglycosides, such as kanamycin and streptomycin, is innate in pediococci and some lactobacilli species, including *L. fermentum* (32, 50). The intrinsic aminoglycoside resistance may be associated with the low level of transmembrane potential or its absence that leads to the impaired uptake of these antibiotics. Moreover, the chromosomal mutations which impact to transmembrane electrical potential, were described in Gram-positive bacteria, while in Gram-negative bacteria the variable efflux systems were identified (32, 66). Furthermore, a high spontaneous mutation rate to resistance to kanamycin and streptomycin in lactobacilli has been reported (67).

In our study, the phenotypic and genotypic resistance do not correspond in many cases since the strains had the MIC values higher that the microbiological cut-off values but did not have the corresponding resistance genes. These findings are consistent with the results reported in other studies regarding AMR of LAB (31, 40–42, 60). The simple explanation could be a mutation and mismatches at the primer annealing site that prevents detection of the target resistance gene (68). The phenotype-genotype discrepancies observed in our study could be also explained by the fact that other resistance genes may exist that were not investigated by us; however the number of the known resistance genes continues to increase. The presence of novel, unknown or unusual resistance determinants should also be considered. Moreover, the resistance might be also acquired through some mutations, for example a high spontaneous mutation rate to resistance to aminoglycosides in lactobacilli has been reported (67). Finally, some LAB species could be intrinsically resistant to certain antimicrobials due to inherent structural and functional features which aid their survival in an environment, but are independent of antibiotic selective pressure and are not spread through horizontal gene transfer. Generally, the regulation of intrinsic resistance is better characterized in Gram-negative bacteria. The available data about AMR in LAB species, are still poorly understood and the further studies should certainly be carried out to clarify this phenomenon (60).

The recent studies have shown the potential of whole genome sequencing (WGS) for define the accurate genotype and link it to the observed phenotypes (55). WGS analysis for AMR allows detection of a much higher number of resistance markers, including the complete set of resistance genes present in isolates as well as the mutations and mobile genetic elements associated with resistance (69). Nevertheless, WGS analysis is still quite expensive as a technique and creates vast amounts of data and requires specialized bioinformatics expertise. Most authors still rely on phenotypic characterization of isolates and PCR-based detection of AMR genes.

The transfer of AMR genes between different LAB species and other bacteria has been well-documented and demonstrated by *in vitro* studies with a filter mating technique, as well as by *in vivo* models of animal rumen and alfalfa plant (29, 70). Moreover, it was shown that AMR genes may be transfer from lactobacilli to *E. faecalis*, which is an inhabitant of the animal and human gut, but also a potential pathogen (70, 71). Although the transferability of the detected resistance markers was not analyzed in our study and specific mobile genetic elements in tested LAB strains were not identified, the nucleotide sequences of the identified AMR genes showed high similarity or even identity to the AMR genes associated with mobile genetic elements, such as transposons and plasmids, described in LAB and other bacteria, even distantly related, and in some cases pathogenic (Supplementary Table 3). This suggests possible acquisition of detected AMR genes from other bacteria. Furthermore, it can be predicted that detected genes are located on mobile genetic elements. Thus, it is important to consider the possibility of further transfer of the detected AMR genes to other bacteria in the gut via horizontal transfer, which poses a serious health risk to animals and humans.

Despite the improved awareness and understanding of AMR of LAB, and the possibility of its spread through the food chain, this safety criterion is not always taking into consideration by researchers (72–74). The results of the current study highlight that the AMR assessment of LAB strains should be the first and key step in considering their applicability and should precede other studies regarding the beneficial effects of the strains, their usefulness or adaptation criteria.

## Conclusion

Concluding, the presence of acquired AMR genes in the tested LAB strains, including genes that were not previously described in this bacterial group, like those found in pathogenic bacteria, confirms that LAB are capable of acquiring resistance determinants via horizontal gene transfer. Importantly, many studies show that such genes can be transferred in both directions. While conjugation is the most common way of dissemination of AMR genes, transformation and transduction may also play an important role in this process, even greater than previously thought (45). Therefore, all strains in this study carrying the acquired AMR genes cannot be considered as safe and should not be used as feed or silage additives. On the other hand, the susceptibility of most of the tested strains to the antibiotics recommended by EFSA make them safe for direct use in agriculture and animal husbandry and thus, worth further exploration.

## Data Availability Statement

The original contributions presented in the study are included in the article/Supplementary Material, further inquiries can be directed to the corresponding author.

## Author Contributions

IS contributed to conception and design of the study. IS, KJ-P, and MG contributed with resources to the study and performed the collection of isolates. IS, EK, and KJ-P conducted the experiments. IS, EK, and MR analyzed the data. IS and EK wrote the draft of the manuscript. MB and MR critically reviewed sections of the manuscript. All authors contributed to manuscript revision, read, and approved the submitted version.

## Conflict of Interest

KJ-P is employed by Prof. Waclaw Dabrowski Institute of Agriculture and Food Biotechnology – State Research Institute, which owns the Industrial Microbial Cultures Collection (KKP). The remaining authors declare that the research was conducted in the absence of any commercial or financial relationships that could be construed as a potential conflict of interest.
