# Supplementary material for: Antimicrobial Susceptibility of Lactic Acid Bacteria Strains of Potential Use as Feed Additives - The Basic Safety and Usefulness Criterion
Source: Front Vet Sci. 2021 Jul 1;8:687071. doi: 10.3389/fvets.2021.687071 (PMC8281277; doi:10.3389/fvets.2021.687071)
Supplement: Supplementary file 1 [file Data_Sheet_1.PDF]

Supplementary Material

Supplementary Table S1.

Characteristics of the lactic acid bacteria strains included in this study.

No.	Strain	Source of isolation
1	*L. buchneri* KKP 2047p*	fermented corn grain
2	*L. buchneri* KKP 3065	fermented corn grain
3	*L. diolivorans* KKP 2036p	fermented cucumber
4	*L. fermentum* KKP 2020	fermented patatoes
5	*L. fermentum* KKP 809	sour milk
6	*L. fermentum* KKP 811	sour milk
7	*L. fermentum* KKP 830	fermented beets
8	*L. fermentum* KKP 843	raw milk
9	*L. fermentum* KKP 1820	fermented beets
10	*L. fermentum* Sieger	grapes
11	*L. brevis* Pap3/4	paprika
12	*L. brevis* Pat1	patison
13	*L. brevis* Solaris	grapes
14	*L. farraginis* E/J	probiotic drink
15	*L. reuteri* KKP 876	gastrointestinal tract of chicken
16	*L. reuteri* KKP 1829	gastrointestinal tract of chicken
17	*L. reuteri* KKP 1837	gastrointestinal tract of chicken
18	*L. agilis* KKP 1832	gastrointestinal tract of chicken
19	*L. agilis* KKP 1834	gastrointestinal tract of chicken
20	*L. salivarius* KKP 1828	gastrointestinal tract of chicken
21	*L. salivarius* KKP 1835	gastrointestinal tract of chicken
22	*L. casei* KKP 824	unknown
23	*L. casei* B3	fermented beets
24	*L. casei* A/J	probiotic drink
25	*L. paracasei* PCM 2639**	unknown
26	*L. rhamnosus* KKP 849	unknown
27	*L. rhamnosus* PMC 489	unknown
28	*L. rhamnosus* B/J	probiotic drink
29	*L. plantarum* KKP 804	fermented cucumber
30	*L. plantarum* KKP 814	fermented cucumber
31	*L. plantarum* KKP 815	fermented cucumber
32	*L. plantarum* KKP 826	unknown
33	*L. plantarum* KKP 831	sauerkraut
34	*L. plantarum* KKP 835	raw milk
35	*L. plantarum* KKP 836	raw milk
36	*L. plantarum* KKP 838	milk
37	*L. plantarum* KKP 840	rumen

38	*L. plantarum* KKP 841	rumen
39	*L. plantarum* KKP 847	raw milk
40	*L. plantarum* KKP 850	unknown
41	*L. plantarum* KKP 870	rumen
42	*L. plantarum* KKP 872	rumen
43	*L. plantarum* KKP 877	gastrointestinal tract of chicken
44	*L. plantarum* KKP 1821	fermented beets
45	*L. plantarum* KKP 1822	fermented beets
46	*L. plantarum* KKP 1831	gastrointestinal tract of chicken
47	*L. plantarum* KKP 1833	gastrointestinal tract of chicken
48	*L. plantarum* KKP 1841	fermented cucumber
49	*L. plantarum* KKP 2021p	silage
50	*L. plantarum* ATCC 8287	green, fermenting Sevillano variety olives
51	*L. plantarum* Pat3	patison
52	*L. plantarum* M1	raspberries
53	*L. plantarum* Regent	grapes
54	*L. plantarum* I/J	probiotic drink
55	*L. delbrueckii* PCM 490	sour grain mash
56	*L. acidophilus* PCM 2499	unknown
57	*L. johnsonii* KKP 878	gastrointestinal tract of chicken
58	*P. pentosaceus* KapA	sauerkraut
59	*P. pentosaceus* Pom7	tomato
60	*P. pentosaceus* AG	gooseberry
61	*P. pentosaceus* MA	raspberries
62	*P. pentosaceus* WN1	grapes
63	*P acidilactici* KKP 1839	fermented cucumber
64	*E. durans* KKP 1586	naturally fermented rye sourdough
65	*E. faecium* TR2	strawberries

*KKP - strains from the Culture Collection of Industrial Microorganisms, located at the Prof. Waclaw Dabrowski Institute of Agricultural and Food Biotechnology - State Research Institute (Warsaw, Poland); **PCM - strains from the Polish Collection of Microorganisms, located at the Institute of Immunology and Experimental Therapy (Wroclaw, Poland)

Supplementary Table S2.

Primers used in this study.

Primer sets and their sequences (5' - 3')	Resistance phenotype	Target genes	Amplicon size (bp)	T_{ann}[1] (ºC)	References
DI GAYACICCIGGICAYRTIGAYTT<br>DII: GCCCARWAIGGRTTIGGIGGIACYTC	TE	genes encoding ribosomal protection proteins	1100	53	(26)
TK1: CAAACTGGGTGAACACAG<br>TL32: CCTGTTCCCTCTGATAAA	TE	*tet*(K), *tet*(L)	1050	45	(27)
tetWF: GAGAGCCTGCTATATGCCAGC<br>tetWR: GGGCGTATCCACAATGTTAAC	TE	*tet*(W)	168	64	(28)
tetMF: ACAGAAAGCTTATTATATAAC<br>tetMR: TGGCGTGTCTATGATGTTCAC	TE	*tet*(M)	656	54	(29)
tetSF: ATCAAGATATTAAGGAC<br>tetSR: TTCTCTATGTGGTAATC	TE	*tet*(S)	573	56	(29)
ermAF: TCTAAAAAGCATGTAAAAGAA<br>ermAR: CTTCGATAGTTTATTAATATTAGT	E, CL	*erm*(A)	645	52	(30)
ermBF: GAAAAGGTACTCAACCAAATA<br>ermBR: AGTAACGGTACTTAAATTGTTTAC	E, CL	*erm*(B)	639	52	(30)
ermCF: TACAAACATAATATAGATAAA<br>ermCR: GCTAATATTGTTTAAATCGTCAAT	E, CL	*erm*(C)	642	52	(30)
msrA/BF: GCAAATGGTGTAGGTAAGACAACT<br>msrA/BR: ATCATGTGATGTAAACAAAAT	E	*msr*(A), *msr*(B)	399	52	(30)
msrCF: GAAAGAAGCGGAAGAACATTA<br>msrCR: AAGCAAAGCAATCGTCAAAC	E	*msr*(C)	354	50	This study
lnuAF: GGTGGCTGGGGGGTAGATGTATTAACTGG<br>lnuAR: GCTTCTTTTGAAATACATGGTATTTTTCGATC	CL	*lnu*(A)	323	55	(31)
catF: TTAGGTTATTGGGATAAGTTA<br>catR: GCATGRTAACCATCACAWAC	CH	*cat*	300	48	(32)
EntcatF: ATGACTTTTAATATTATTRAWTT<br>EntcatR: TCATYTACMYTATSAATTATAT	CH	*Entcat*	684	50	(32)
blaF: CATARTTCCGATAATASMGCC<br>blaR: CGTSTTTAACTAAGTATSGY	A	*blaZ*	297	51	(32)
aph(3")-IIIaF: GGCTAAAATGAGAATATCACCGG<br>aph(3")-IIIaR: CTTTAAAAAATCATACAGCTCGCG	K	*aph*(3')-*IIIa*	523	55	(33)
aac(6')aph(2")F: CCAAGAGCAATAAGGGCATA<br>aac(6')aph(2")R: CACTATCATAACCACTACCG	GM, S, K	*aac*(6')*aph*(2")	220	48	(34)

ant(6)F: ACTGGCTTAATCAATTTGGG<br>ant(6)R: GCCTTTCCGCCACCTCACCG	S	*ant*(6)	597	58	(35)
strA-strBF: TATCTGCGATTGGACCCTCTG<br>strA-strBR: CATTGCTCATCATTTGATCGGCT	S	*str*(A), *str*(B)	538	60	(36)
aadAF: ATCCTTCGGCGCGATTTTG<br>aadAR: GCAGCGCAATGACATTCTTG	S	*aad*(A)	198	48	

[1] T_{ann} - annealing temperature.
TE, tetracycline; E, erythromycin; CL, clindamycin; CH, chloramphenicol; A, ampicillin; K, kanamycin; GM, gentamicin; S, streptomycin.

Supplementary Table S3.

Analysis of the obtained nucleotide sequences using the GenBank and CARD database.

<table>
<tr><th rowspan="2">Strain</th><th colspan="4">GenBank analysis</th><th>CARD analysis</th></tr>
<tr><th>Resistance gene detected in this study</th><th>Description</th><th>Identity</th><th>GenBank accession number</th><th>Resistance gene and description</th></tr>
<tr><td rowspan="4">L. agilis KKP 1834</td><td rowspan="2">aph(3”)-IIIa</td><td>Enterococcus faecalis strain T90-6 plasmid T90-6</td><td>100%</td><td>CP069129</td><td rowspan="2">aph(3”)-IIIa aminoglycoside phosphotransferase</td></tr>
<tr><td>Streptococcus pneumoniae integrative and conjugative element ICESpnSPN8332, isolate SPN8332</td><td>99.8%</td><td>HG799498</td></tr>
<tr><td>Msr family ABC-F type ribosomal protection protein</td><td>Enterococcus faecium strain VVEswe-R</td><td>100%</td><td>HQ651922</td><td rowspan="2">msr(C) ABC-F ATP-binding cassette ribosomal protection</td></tr>
<tr><td>msr(C)</td><td>Enterococcus faecium strain VRE33012</td><td>99.7%</td><td>JAEKBB01000 0043</td></tr>
<tr><td rowspan="2">L. diolivorans KKP 2036p</td><td rowspan="2">aph(3”)-IIIa</td><td>Campylobacter jejuni strain T1-21 plasmid pcjDM</td><td>100%</td><td>CP013117</td><td rowspan="2">aph(3”)-IIIa aminoglycoside phosphotransferase</td></tr>
<tr><td>Streptococcus pneumoniae integrative and conjugative element ICESpnSPN8332, isolate SPN8332</td><td>100%</td><td>HG799498</td></tr>
<tr><td rowspan="4">L. salivarius KKP 1835</td><td rowspan="2">tet(M)</td><td>Neisseria gonorrhoeae strain NG196 plasmid p1</td><td>99.5%</td><td>CP043872</td><td rowspan="2">tet(M) tetracycline-resistant ribosomal protection protein</td></tr>
<tr><td>Streptococcus pneumoniae integrative and conjugative element ICE6BST90, isolate IC161</td><td>99.7%</td><td>HG799499</td></tr>
<tr><td rowspan="2">str(B)</td><td>Escherichia coli H20 strain MIN6 plasmid pMUB-MIN6-1</td><td>99%</td><td>CP069693</td><td rowspan="2">aph(6)-Id aminoglycoside phosphotransferase</td></tr>
<tr><td>Enterobacter hormaechei strain CM18-242-2 plasmid pCM18-242-2</td><td>99%</td><td>CP050507</td></tr>
<tr><td rowspan="2">L. acidophilus PCM 2499</td><td rowspan="2">tet(K)</td><td>Staphylococcus aureus plasmid pT181 tetracycline efflux protein (tet(K)) gene</td><td>100%</td><td>NG034604</td><td rowspan="2">tet(K) major facilitator superfamily (MFS) antibiotic efflux pump</td></tr>
<tr><td>Staphylococcus aureus subsp. aureus strain 11819-97 plasmid pT49</td><td>100%</td><td>KM281803</td></tr>
<tr><td rowspan="2">E. durans KKP 1586</td><td rowspan="2">erm(B)</td><td>Lactobacillus reuteri plasmid pTE80 rRNA methylase regulatory protein and rRNA methylase (erm) genes</td><td>99.3%</td><td>AF080450</td><td rowspan="2">erm(B) 23S ribosomal RNA methyltransferase</td></tr>
<tr><td>Streptococcus pneumoniae transposon Tn1545 containing erm gene for rRNA methylase</td><td>99.7%</td><td>X52632</td></tr>
<tr><td rowspan="2">L. plantarum KKP 2021p</td><td rowspan="2">erm(B)</td><td>Lactobacillus reuteri plasmid pTE80 rRNA methylase regulatory protein and rRNA methylase (erm) genes</td><td>98.3%</td><td>AF080450</td><td rowspan="2">erm(B) 23S ribosomal RNA methyltransferase</td></tr>
<tr><td>Streptococcus pneumoniae transposon Tn917-like LP-1, ermB genes for leader peptide, adenine-N6 methyltransferase</td><td>98.3%</td><td>AB111455</td></tr>
<tr><td rowspan="2">L. plantarum KKP 870</td><td rowspan="2">lnu(A)</td><td>Lactobacillus plantarum strain PA18 plasmid pR18</td><td>98.5%</td><td>JN601038</td><td rowspan="2">lnu(A) lincosamide nucleotidyltransferase</td></tr>
<tr><td>Staphylococcus aureus strain C5425 plasmid pUR5425</td><td>98.5%</td><td>JQ861958</td></tr>
</table>

E. faecium TR2	*tet*(M)	*Enterococcus faecium* strain TC 6 transposon Tn6086	99.7%	HM636636	*tet*(M) tetracycline-resistant ribosomal protection protein
		Clostridium difficile strain CD2386 transposon Tn916-like TetM protein (tetM) gene	99.9%	JN846696	
	lnu(A)	*Lactobacillus pentosus* strain F03 plasmid pF03-2	99.6%	KJ868089	*lnu*(A) lincosamide nucleotidyltransferase
		Staphylococcus aureus strain C5425 plasmid pUR5425	99.2%	JQ861958	
	msr(C)	*Enterococcus faecium* strain VRE33012	98.9%	JAEKBB010000043	*msr*(C) ABC-F ATP-binding cassette ribosomal protection

Supplementary Table S4.

The nucleotide sequences of the resistance genes detected in the tested lactic acid bacteria strains.

Strain	Gene	Sequence
E. faecium TR2	*msr*(C)	TTTTCTTTAGGTAAAAAAATCGCGATTGTCGGTGAAAATGGTTCAGGTA<br>AGACAACCTTATTAGAACATATCCGCAAACAAGGAGAAGGAATCCTTC<br>TCTCTCCGAAAGTAAGCTTTCAAGTATATCAGCAAAAGGGTTATCAAAT<br>GACATCTGAAGAATCCATCATTCGTTTTGTCATGASACAAACAGAGTTT<br>TCAGAATCGCTTGTCCGTAGTTTGCTGAATCACTTAGGGTTTGCTCAGG<br>AAACTCTGACKAAACCGTTATGTACATTAAGTGGGGGA
L. agilis KKP 1834	*msr*(C)	ATCCTTCTCTCTCCGAAAGTAAGCTTTCAAGTATATCAGCAAAAGGGTT<br>ATCAAATGACATCTGAAGAATCCATCATTCGTTTTGTCATGAGACAAAC<br>AGAGTTTTCAGAATCGCTTGTCCGTAGTTTGCTGAATCACTTAGGGTTT<br>GCTCAGGAAACTCTGACGAAACCGTTATGTACATTAAGTGGGGGAGAA<br>GCGACCCGTCTGACGATTGCTTTGCTTTTTACTAAGCCAAGTAATGTGT<br>TGCTGTTAGATGAACCGACTAATTTTATTGATATGGCAACGATCGAAGC<br>TTTAGAGAAGCTAATGCAAATATATCCGGGAACGATTTTGTTTA
L. plantarum KKP 2021p	*erm*(B)	ACCGATACCGTTTACGAAATTGGAACAGGTAAAGGGCATTTAACGACG<br>AAACTGGCTAAAATAAGTAAACAGGTAACGTCTATTGAATTAGACAGT<br>CATCTATTCAACTTATCGTCAGAAAAATTAAAACTGAACATTCGTGTCA<br>CTTTAATTCACCAAGATATTCTACAGTTTCAATTCCCTAACAAACAGAG<br>GTATAAAATTGTTGGGAATATTCCTTACCATTTAAGCACACAAATTATT<br>AAAAAAGTGGTTTTTGAAAGCCATGCGTCTGACATCTATCTGATTGTTG<br>AAGAAGGATTCTACAAGCGTACCTTGGATATTCACCGAACACTAGGGT<br>TGCTCTTGCACACTCAAGTCTCGATTCAGCAATTGCTTAAGCTGCCAGC<br>GGAATGCTTTCATCCTAAACCAAAAGTAAACAGTGTCTTAATAAAACTT<br>ACCCGCCATACCACAGATGTTCCAGATAAATATTGGAARCTATATACGT<br>ACTTTGTTTCAAAATGGGTCAATCGAGAATATCGTCAACTGTTTACTAA<br>AAATCAGTTTCATCAAGCAATGAAACACGCCAAAGTAAACAATTTAA
E. durans KKP 1586	*erm*(B)	ACCGTTTACGAAATTGGAACAGGTAAAGGGCATTTAACGACGAAACTG<br>GCTAAAATAAGTAAACAGGTAACGTCTATTGAATTAGACAGTCATCTAT<br>TCAACTTATCGTCAGAAAAATTAAAACTGAATACTCGTGTCACTTTAAT<br>TCACCAAGATATTCTACAGTTTCAATTCCCTAACAAACAGAGGTATAAA<br>ATTGTTGGGAATATTCCTTACCATTTAAGCACACAAATTATTAAAAAAG<br>TGGTTTTTGAAAGCCGTGCGTCTGACATCTATCTGATTGTTGAAGAAGG<br>ATTCTACAAGCGTACCTTGGATATTCACCGAACACTAGGGTTGCTCTTG<br>CACACTCAAGTCTCGATTAAGCAATTGCTTAAGCTGCCAGCTGAATGCT<br>TTCAACCTAAACCAAAAGTAAACAGTGCCTTAATAAAACTTACCCGCC<br>ATACCACAGATGTTCCAGATAAATATTGGAAACTATATACGTACTTTGT<br>TTCAAAATGGGTCAATCGAGAATATCGTCAACTGTTTACTAAAAATCAG<br>TTTCATCAAGCAATGAAATACGCCAAAGTAAACAATTTAG
L. plantarum KKP 870	*lnu*(A)	GATTTTGACGCATCACACACTCAAAAAGTTATACAAAAATTAGAAGAT<br>ATCGGATACAAAATAGAAGTTGATTGGATGCCTTCACGTATGGAACTC<br>AAGCATGAAGAATATGGGTATTTAGATATTCATCCTATAAATCTAAATG<br>ATGATGGATCAATTACCCAAGCAAACCCAGAAGGTGGTAATTATGTTTT<br>CCAAAATGACTGGTTTTCAGAAACTAATTACAAAGATCGAAAAATACC<br>ATGTATTTCAAAAGAAGC

E. faecium TR2	*lnu*(A)	CACACTCAAAAAGTTATACAAAAATTAGAAGATATCGGATACAAAATAGAAGTTGATTGGATGCCTTCACGTATGGAACTCAAGCATGAAGAATATGGGTATTTAGATATTCATCCTATAAATCTAAATGATGATGGATCAATTACCCAAGCAAACCCAGAAGGTGGTAATTATGTTTTCCAAAATGACTGGTTTTCAGAAACTAATTACAAAGATCGAAAAATACCATGTATTTCAAAAGAAG
L. agilis KKP 1834	*aph*(3'')-*IIIa*	GATACGGAAGGAATGTCTCCTGCTAAGGTATATAAGCTGGTGGGAGAAAATGAAAACCTATATTTAAAAATGACGGACAGCCGGTATAAAGGGACCACCTATGATGTGGAACGGGAAAAGGACATGATGCTATGGCTGGAAGGAAAGCTGCCTGTTCCAAAGGTCCTGCACTTTGAACGGCATGATGGCTGGAGCAATCTGCTCATGAGTGAGGCCGATGGCGTCCTTTGCTCGGAAGAGTATGAAGATGAACAAAGCCCTGAAAAGATTATCGAGCTGTATGCGGAGTGCATCAGGCTCTTTCACTCCATCGACATATCGGATTGTCCCTATACGAATAGCTTAGACAGCCGCTTAGCCGAATTGGATTACTTACTGAATAACGATCTGGCCGATGTGGATTGCGAAAACTGGGAAGAAGACACTCCATTTAAAGATCCGCGCGAGCTGTATGTTTTTTTAAAGA
L. diolivorans KKP 2036p	*aph*(3'')-*IIIa*	GATACGGAAGGAATGTCTCCTGCTAAGGTATATAAGCTGGTGGGAGAAAATGAAAACCTATATTTAAAAATGACGGACAGCCGGTATAAAGGGACCACCTATGATGTGGAACGGGAAAAGGACATGATGCTATGGCTGGAAGGAAAGCTGCCTGTTCCAAAGGTCCTGCACTTTGAACGGCATGATGGCTGGAGCAATCTGCTCATGAGTGAGGCCGATGGCGTCCTTTGCTCGGAAGAGTATGAAGATGAACAAAGCCCTGAAAAGATTATCGAGCTGTATGCGGAGTGCATCAGGCTCTTTCACTCCATCGACATATCGGATTGTCCCTATACGAATAGCTTAGACAGCCGCTTAGCCGAATTGGATTACTTACTGAATAACGATCTGGCCGATGTGGATTGCGAAAACTGGGAAGAAGACACTCCATTTAAAGATCCGCGCGAGCTGTAT
L. salivarius KKP 1835	*str*(B)<br><br>(*aph(6)-Id*)	ATGCCGCCTGTTTTTCCTGCTCATTGGCACGTTTCGCAACCTGTTCTCATTGCGGACACCTTTTCCAGCCTCGTTTGGAAAGTTTCATTGCCAGACGGGACTCCTGCAATCGTCAAGGGATTGAAACCTATAGAAGACATTGCTGATGAACTGCGCGGGGCCGACTATCTGGTATGGCGCAATGGGAGGGGAGCAGTCCGGTTGCTCGGTCGTGAGAACAATCTGATGTTGCTCGAATATGCCGGGGAGCGAATGCTCTCTCACATCGTTGCCGAGCACGGCGACTACCAGGCGACCGAAATTGCAGCGGAACTAATGGCGAAGCTGTATGCCGCATCTGAGGAACCCCTGCCTTCTGCCCTTCTCCCGATCCGGGATCGCTTTGCAGCTTTGTTTCAGCGGGCGCGCGATGATCAAAACGCAGGTTGTCAAACTGACTACGTCCACGCGGCGATTATAGCCGATCAAATGAT
L. salivarius KKP 1835	*tet*(M)	AATCCCCTCCCTCTGCTGCAAACGACTGTTGAACCGAGCAAACCTCAACAAAGGGAAATGTTACTTGATGCACTTTTAGAAATCTCCGACAGTGACCCGCTTCTGCGATATTATGTGGATTCTGCGACACATGAAATCATACTTTCTTTCTTAGGGAAAGTACAAATGGAAGTGACTTGTGCTCTGCTGCAAGAAAAGTATCATGTGGAGATAGAAATAAAAGAGCCTACAGTCATTTATATGGAAAGACCGTTAAAAAAAGCAGAGTATACCATTCACATCGAAGTTCCACCGAATCCTTTCTGGGCTTCCATTGGTCTATCTGTAGCACAGCTTCCATTAGGGAGCGGAGTACAGTATGAGAGCTCGGTTTCTCTTGGATACTTAAATCAATCTTTTCAAAATGCAGTTATGGAGGGGATACGCTATGGCTGTGAACAAGGATTGTATGGTTGGAATGTGACGGACTGTAAAATCTGTTTTAAGTATGGCTTATACTATAGCCCTGTTAGTACCCCAGCAGATTTTCGGATGCTTG

		CTCCTATTGTATTGGAACAAGTCTTAAAAAAAGCTGGAACAGAATTGTT<br>AGAGA
E. faecium<br>TR2	*tet*(M)	TTATCAGTTTTAGATGGGGCAATTCTACTGATTTCTGCAAAAGATGGCG<br>TACAAGCACAAACTCGTATATTATTTCATGCACTTAGGAAAATGGGGAT<br>TCCCACAATCTTTTTTATCAATAAGATTGACCAAAATGGAATTGATTTA<br>TCAACGGTTTATCAGGATATTAAAGAGAAACTTTCTGCCGAAATTGTAA<br>TCAAACAGAAGGTAGAACTGTATCCTAATGTGTGTGTGACGAACTTTAC<br>CGAATCTGAACAATGGGATACGGTAATAGAGGGAAACGATGACCTTTT<br>AGAGAAATATATGTCCGGTAAATCATTAGAAGCATTGGAACTCGAACA<br>AGAGGAAAGCATAAGATTTCAGAATTGCTCCTTGTACCCTGTTTATCAT<br>GGAAGCGCAAAAAGCAACATAGGGATTGAGCAGCTTATAGAAGTGATA<br>ACGAATAAATTTTATTCATCAACATACAGAAAGAAGTCTGAACTTTGCG<br>GAAATGTCTTCAAAATTGAATATTCGGAAGAAAGACAACGTCTTGCAT<br>ATGTACGCCTTTATGGCGGAATCCTGCATTTGCGGGATTCGGTTAGAAT<br>ATCGGAAAAGGAAAAAATAAAAATTACAGAAATGTATACTTCAATAAA<br>TGGTGAATTATGTAAAATTGATAAGGCTTATTCCGGGGAAATTGTTATT<br>TTGCAAAATGAGTTTTTGAAGCTAAATAGTGTTCTTGGAGATACAAAGC<br>TATTGCCACAGAGAGAGAGAATTGAAAATCCGCTCCCTCTGCTGCAAA<br>CAACTGTTGAACCGAGCAAACCTCAACAAAGGGAAATGTTACTTGATG<br>CACTTTTAGAAATCTCCGACAGTGACCCGCTTCTACAATATTATGTGGA<br>TTCTACGACACATGAAATCATACTTTCTTTCTTAGGGAAAGTACAAATG<br>GAAGTGACTTGTGCTCTATTGCAAGAAAAGTATCATGTGGAGGTAAAA<br>ATAAAAAAGCCTACAGTCATTTATATGGAAAGACCGTTAAAAAAAGCA<br>GAGTATACCATTCACATC
L. acidophilus<br>PCM 2499	*tet*(K)	AGTATATGGAAAATTATCTGATTATATAAATATAAAAAAATTGTTAATT<br>ATTGGTATTAGTTTGAGCTGTCTTGGTTCATTGATTGCTTTTATTGGTCA<br>CAATCACTTTTTTATTTTGATTTTTGGTAGGTTAGTACAAGGAGTAGGAT<br>CTGCTGCATTCCCTTCACTGATTATGGTGGTTGTAGCTAGAAATATTAC<br>AAGAAAAAAACAAGGCAAAGCCTTTGGTTTTATAGGATCAATTGTAGC<br>TTTAGGTGAAGGGTTAGGTCCTTCAATAGGGGGAATAATAGCACATTAT<br>ATTCATTGGTCTTACCTACTTATACTTCCTATGATTACAATAGTAACTAT<br>ACCTTTTCTTATTAAAGTAATGGTACCTGGTAAATCAACAAAAAATACA<br>TTAGATATCGTAGGTATTGTTTTAATGTCTATAAGTATTATATGTTTTAT<br>GTTATTTACGACAAATTATAATTGGACTTTTTTAATACTCTTCACAATCT<br>TTTTTGTGATTTTTATTAAACATATTTCAAGAGTTTCTAACCCTTTTATT<br>AATCCTAAACTAGGGAAAAACATTCCGTTTATGCTTGGTTTGTTTTCTG<br>GTGGGCTAATATTTTCTATAGTAGCTGGTTTTATATCAATGGTGCCTTAT<br>ATGATGAAAACTATTTATCATGTAAATGTAGCGACAATAGGTAATAGT<br>GTTATTTTTCCTGGAACCATGAGTGTTATTGTTTTTGGTTATTTTGGTGG<br>TTTTTTAGTGGATAGAAAAGGATCATTATTTGTTTTTATTTTAGGATCAT<br>TGTCTATCTCTATAAGTTTTTTAACTATTGCATTTTTTGTTGAGTTTAGTA<br>TGTGGTTGACTACTTTTATGTTTATATTTGTTATGGGCGGATTATCTTTT<br>ACTAAAACAGTTATATCAAAAATAGTATCAAGTAGTCTTTCTGAAGAA<br>GAAGTTGCTTCTGGAATGAGTTTGCTAAATTTCACAAGTTTTT

Supplementary Table S5.

Distribution of MICs of tested antibiotics among various LAB strains (n=65).

Number	Strain	MIC (mg/L)[1]							
		GM[2]	K	TE	CH	A	E	CL	S
**Microbiological cut-off values (mg/L) proposed by EFSA for obligate heterofermentative *Lactobacillus***									
		16	64(32)[3]	8	4	2	1	4(1)	64
**1**	*L. buchneri* KKP 2047p	4	**128**	16[4]	4	1	0,25	0,5	64
**2**	*L. buchneri* KKP 3065	0,5	16	16[4]	4	1	0,03	0,03	16
**3**	*L. diolivorans* KKP 2036p	8	**128**	**16**	2	2	0,25	0,125	**128**
**4**	*L. fermentum* KKP 2020	2	32	**16**	4	0,5	0,25	0,25	32
**5**	*L. fermentum* KKP 1820	8	64	4	2	0,125	0,25	0,03	16
**6**	*L. fermentum* KKP 809	1	16	4	2	≤0,015	0,125	≤0,015	8
**7**	*L. fermentum* KKP 811	4	64	4	2	0,06	0,06	0,03	32
**8**	*L. fermentum* KKP 830	8	64	**16**	2	1	0,25	0,25	64
**9**	*L. fermentum* KKP 843	2	64	4	2	0,03	0,06	≤0,015	32
**10**	*L. fermentum* Sieger	16	**128**	4	4	0,125	0,125	0,03	32
**11**	*L. brevis* Pap3/4	2	64	**16**	4	0,25	0,125	0,25	64
**12**	*L. brevis* Pat1	0,5	16	**16**	4	2	0,5	≤0,015	8
**13**	*L. brevis* Solaris	1	16	**16**	4	1	0,25	0,5	16
**14**	*L. farraginis* E/J	0,5	8	**16**	4	0,125	0,03	0,03	8
**Microbiological cut-off values (mg/L) proposed by EFSA for *Lactobacillus reuteri***									
		8	64	32(16)	4	2	1	4(1)	64
**15**	*L. reuteri* KKP 876	4	32	16	4	1	0,25	0,25	32
**16**	*L. reuteri* KKP 1829	≤0,125	4	1	1	0,5	0,125	≤0,015	4
**17**	*L. reuteri* KKP 1837	4	64	2	2	1	0,25	0,03	16
**Microbiological cut-off values (mg/L) proposed by EFSA – facultative heterofermentative *Lactobacillus***									
		16	64	8	4	4	1	4(1)	64
**18**	*L. agilis* KKP 1832	4	32	0,25	1	0,25	≤0,015	0,06	4
**19**	*L. agilis* KKP 1834	**32**	**≥256**	0,25	4	1	**≥8**	1	**≥256**
**20**	*L. salivarius* KKP 1828	16	**128**	1	4	0,25	0,125	0,125	**128**
**21**	*L. salivarius* KKP 1835	8	**128**	**16**	4	2	0,125	0,03	**128**

	**Microbiological cut-off values (mg/L) proposed by EFSA for *Lactobacillus casei/paracasei***								
		32	64	4	4	4	1	4(1)	64
**22**	*L. casei* B3	8	64	1	4	1	0,125	0,125	32
**23**	*L. casei* A/J	32	256	1	4	0,5	0,06	4	64
**24**	*L. casei* KKP 824	2	32	1	4	4	0,06	0,25	16
**25**	*L. paracasei* ssp. *paracasei* PCM 2639	4	32	1	4	0,5	0,125	0,25	16
	**Microbiological cut-off values (mg/L) proposed by EFSA for *Lactobacillus rhamnosus***								
		16	64	8	4	4	1	4(1)	32
**26**	*L. rhamnosus* PCM489	2	16	1	4	1	0,25	0,5	≤0,5
**27**	*L. rhamnosus* KKP 849	4	**128**	1	**8**	1	0,5	0,5	32
**28**	*L. rhamnosus* B/J	**32**	**128**	1	4	0,5	0,125	0,5	32
	**Microbiological cut-off values (mg/L) proposed by EFSA for *Lactobacillus plantarum/pentosus***								
		16	64	32	8	2	1	4(2)	n.r.
**29**	*L. plantarum* KKP 804	4	64	32	4	**≥8**	0,25	4	n.r.
**30**	KKP 814	2	64	16	4	1	0,25	2	n.r.
**31**	KKP 815	8	**128**	16	8	1	0,25	2	n.r.
**32**	KKP 826	4	64	16	8	0,25	0,25	0,5	32
**33**	KKP 835	8	**≥256**	16	8	2	0,25	1	n.r.
**34**	KKP 850	2	16	16	8	0,25	0,25	0,06	n.r.
**35**	KKP 870	16	**≥256**	32	8	2	0,25	4	n.r.
**36**	KKP 872	16	**≥256**	16	8	2	0,25	4	n.r.
**37**	KKP 877	4	64	16	8	0,25	0,25	0,5	n.r.
**38**	KKP 2021p	4	**128**	16	8	1	0.25	4	n.r.
**39**	KKP 1833	2	32	8	2	2	0,125	0,5	n.r.
**40**	KKP 1821	4	**128**	16	4	1	0,25	0,5	n.r.
**41**	KKP 1822	8	**128**	16	8	1	0,25	0,5	n.r.
**42**	KKP 836	1	32	32	4	0,125	0,125	0,125	n.r.
**43**	ATTC 8287	8	**128**	16	8	2	0,5	2	n.r.
**44**	KKP 838	4	64	32	8	0,25	0,25	0,5	n.r.
**45**	KKP 840	2	32	32	4	0,125	0,125	0,125	n.r.
**46**	KKP 841	1	32	16	4	0,125	0,125	1	n.r.
**47**	KKP 847	1	32	16	4	0,125	0,125	≤0,015	n.r.
**48**	KKP 1841	1	16	8	4	0,125	0,125	0,25	n.r.

**49**	KKP 831	1	32	16	8	0,125	0,125	0,5	n.r.
**50**	Pat3	8	64	16	4	0,125	0,125	0,125	n.r.
**51**	KKP 1831	2	32	16	4	0,25	0,125	≤0,015	n.r.
**52**	M1	8	64	16	8	0,125	0,25	0,5	n.r.
**53**	Regent	8	64	16	4	0,125	0,25	0,25	n.r.
**54**	I/J	1	4	0,5	8	0,06	1	0,25	n.r.
**Microbiological cut-off values (mg/L) proposed by EFSA for obligate homofermentative *Lactobacillus***									
		16	16	4	4	2(1)	1	4(1)	16
**55**	*L. delbrueckii* PCM 490	4	**32**	2	2	0,06	0,06	0,06	8
**Microbiological cut-off values (mg/L) proposed by EFSA for *Lactobacillus acidophilus* group**									
		16	64	4	4	1	1	4(1)	16
**56**	*L. acidophilus* PCM 2499	4	16	**32**	2	0,25	1	0,125	**32**
**57**	*L. johnsonii* KKP 878	4	64	**16**	**8**	0,125	0,25	0,5	**32**
**Microbiological cut-off values (mg/L) proposed by EFSA for *Pediococcus* spp.**									
		16	64	8	4	4	1	1	64
**58**	*P. pentosaceus* KapA	4	**128**	**16**	4	2	0,25	0,03	**128**
**59**	*P. pentosaceus* Pom7	4	64	**16**	2	1	0,25	0,03	64
**60**	*P. pentosaceus* AG	16	**128**	**16**	4	2	0,5	0,03	**128**
**61**	*P. pentosaceus* MA	16	**≥256**	**16**	4	2	0,25	0,03	64
**62**	*P. pentosaceus* WN1	8	64	**16**	4	1	0,5	0,03	**128**
**63**	*P. acidilactici* KKP 1839	4	**128**	**16**	4	2	0,25	0,03	**128**
**Microbiological cut-off values (mg/L) proposed by EFSA for *Enterococcus* spp.**									
		32	1024	4	16	2	4	4	128
**64**	*E. durans* KKP 1586	16	64	0,5	8	0,25	**≥8**	**≥8**	128
**65**	*E. faecium* TR2	32	128	**32**	2	0,125	**≥8**	4	128

[1] MICs higher than EFSA cut-off values in bold; [2] GM – gentamicin, K – kanamycin, TE – tetracycline, CH – chloramphenicol, A – ampicillin, E – erythromycin, CL – clindamycin, S – streptomycin; [3] the previous EFSA proposed cut-off values (2012) are given in brackets; [4] *L. buchneri* the cut-off for tetracycline is 128; KKP - strains from the Culture Collection of Industrial Microorganisms; PCM - strains from The Polish Collection of Microorganisms; n.r. – not required.
